# The complete chloroplast genome sequence of *Cautleya gracilis*

**DOI:** 10.1080/23802359.2019.1688713

**Published:** 2019-11-12

**Authors:** Yunqing Li, Yi Wang

**Affiliations:** Laboratory of Forest Plant Cultivation and Utilization, Yunnan Academy of Forestry, Kunming, People’s Republic of China

**Keywords:** *Cautleya gracilis*, chloroplast, Illumina sequencing, phylogenetic analysis

## Abstract

The first complete chloroplast genome (cpDNA) sequence of *Cautleya gracilis* was determined from Illumina HiSeq pair-end sequencing data in this study. The cpDNA is 164,001 bp in length, contains a large single-copy region (LSC) of 89,271 bp and a small single-copy region (SSC) of 15,984 bp, which were separated by a pair of inverted repeats (IR) regions of 29,373 bp. The genome contains 131 genes, including 85 protein-coding genes, 8 ribosomal RNA genes, and 38 transfer RNA genes. The overall GC content of the whole genome is 36.1% and the corresponding values of the LSC, SSC, and IR regions are 33.8, 29.4, and 41.3%, respectively. Further phylogenomic analysis showed that *C. gracilis* close to genus *Curcuma* in family Zingiberaceae.

*Cautleya gracilis* is the species of the genus *Cautleya* within the family Zingiberaceae. It distributes in Tibet, Yunnan, and Sichuan of China, India, and Nepal. *Cautleya gracilis* usually grows in the wet valley, sometimes epiphytic to trees (Tang et al. 2001). The extracts of *C. gracilis* showed anticancer activity on human medullary carcinoma cells. The extracts from *C. gracilis* had a strong inhibitory effect on tumour cell growth, disrupting the tumour spheroids, and induced tumour cell apoptosis (Li et al. 2008). Therefore, *C. gracilis* has huge potential medicinal value. However, there have been no genomic studies on *C. gracilis*.

Herein, we reported and characterized the complete *C. gracilis* plastid genome (MN539264). One *C. gracilis* individual (specimen number: 5309270808) was collected from Puwen, Yunnan Province of China (23°20′9″ N, 99°14′16″ E). The specimen is stored at Yunnan Academy of Forestry Herbarium, Kunming, China and the accession number is YAFM20180726. DNA was extracted from its fresh leaves using DNA Plantzol Reagent (Invitrogen, Carlsbad, CA, USA).

Paired-end reads were sequenced by using Illumina HiSeq system (Illumina, San Diego, CA, USA). In total, about 25.3 million high-quality clean reads were generated with adaptors trimmed. Aligning, assembly, and annotation were conducted by CLC de novo assembler (CLC Bio, Aarhus, Denmark), BLAST, GeSeq (Tillich et al. 2017), and Geneious version 11.0.5 (Biomatters Ltd, Auckland, New Zealand). To confirm the phylogenetic position of *C. gracilis*, other nine species of family *Zingiberaceae* from NCBI were aligned using MAFFT version 7 (Katoh and Standley 2013). The Auto algorithm in the MAFFT alignment software was used to align the eight complete genome sequences and the G-INS-i algorithm was used to align the partial complex sequecnces and maximum likelihood (ML) bootstrap analysis was conducted using RAxML (Stamatakis 2006); bootstrap probability values were calculated from 1000 replicates. *Musella lasiocarpa* (KY807173) and *Musa balbisiana* (KT595228) were served as the out-group.

The complete *C. gracilis* plastid genome is a circular DNA molecule with the length of 164,001 bp, contains a large single-copy region (LSC) of 89,271 bp and a small single-copy region (SSC) of 15,984 bp, which were separated by a pair of inverted repeats (IR) regions of 29,373 bp. The overall GC content of the whole genome is 36.1%, and the corresponding values of the LSC, SSC, and IR regions are 33.8, 29.4, and 41.3%, respectively. The plastid genome contained 131 genes, including 85 protein-coding genes, 8 ribosomal RNA genes, and 38 transfer RNA genes. Further, phylogenomic analysis showed that *C. gracilis* close to the genus *Curcuma* in family *Zingiberaceae* (Figure 1). The determination of the complete plastid genome sequences provided new molecular data to illuminate the *Zingiberaceae* evolution.

**Figure 1.**
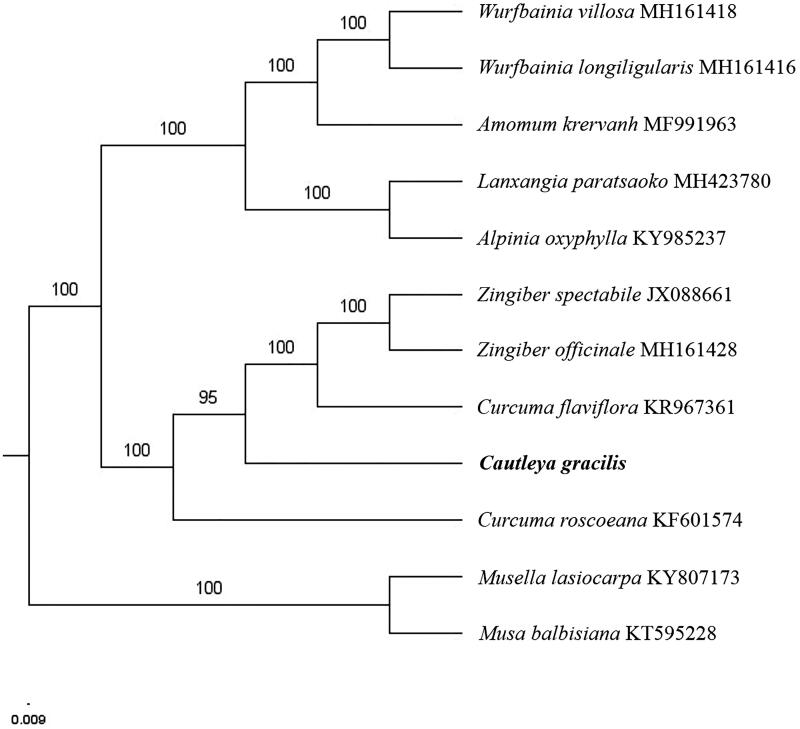
The maximum-likelihood tree based on the 10 chloroplast genomes of *Zingiberaceae*. The bootstrap value based on 1000 replicates is shown on each node.
